# A high reticulocyte count is a risk factor for the onset of metabolic dysfunction-associated steatotic liver disease: Cross-sectional and prospective studies of data of 310,091 individuals from the UK Biobank

**DOI:** 10.3389/fphar.2024.1281095

**Published:** 2024-07-01

**Authors:** Peng-Cheng Ma, Qi-Mei Li, Rui-Ning Li, Chang Hong, Hao Cui, Zi-Yong Zhang, Yan Li, Lu-Shan Xiao, Hong Zhu, Lin Zeng, Jun Xu, Wei-Nan Lai, Li Liu

**Affiliations:** ^1^ School of Public Health, Southern Medical University, Guangzhou, China; ^2^ School of Health Management, Southern Medical University, Guangzhou, China; ^3^ Nanfang Hospital, Southern Medical University, Guangzhou, China; ^4^ Guangdong Provincial Key Laboratory of Viral Hepatitis Research, Hepatology Unit and Department of Infectious Diseases, Nanfang Hospital, Southern Medical University, Guangzhou, China; ^5^ Big Data Center, Nanfang Hospital, Southern Medical University, Guangzhou, China; ^6^ Department of Rheumatology and Immunology, Nanfang Hospital, Southern Medical University, Guangzhou, China

**Keywords:** metabolic dysfunction-associated steatotic liver disease (MASLD), reticulocyte, risk factor, UK Biobank, NAFLD

## Abstract

**Background and Aims:** Metabolic dysfunction-associated steatotic liver disease (MASLD) poses a considerable health risk. Nevertheless, its risk factors are not thoroughly comprehended, and the association between the reticulocyte count and MASLD remains uncertain. This study aimed to explore the relationship between reticulocyte count and MASLD.

**Methods:** A total of 310,091 individuals from the UK Biobank were included in this cross-sectional study, and 7,316 individuals were included in this prospective study. The cross-sectional analysis categorized reticulocyte count into quartiles, considering the sample distribution. Logistic regression models examined the connection between reticulocyte count and MASLD. In the prospective analysis, Cox analysis was utilized to investigate the association.

**Results:** Our study findings indicate a significant association between higher reticulocyte count and an elevated risk of MASLD in both the cross-sectional and prospective analyses. In the cross-sectional analysis, the adjusted odds ratios (ORs) of MASLD increased stepwise over reticulocyte count quartiles (quartile 2: OR 1.22, 95% CI 1.17–1.28, *p* < 0.001; quartile 3: OR 1.44; 95% CI 1.38–1.51, *p* < 0.001; quartile 4: OR 1.66, 95% CI 1.59–1.74, *p* < 0.001). The results of prospective analyses were similar.

**Conclusion:** Increased reticulocyte count was independently associated with a higher risk of MASLD. This discovery offers new insights into the potential of reticulocytes as biomarkers for MASLD.

## Introduction

The increasing prevalence of obesity has contributed to nonalcoholic fatty liver disease (NAFLD) becoming highly prevalent liver diseases, affecting up to 38% of the global population (Targher et al., 2024). However, recently, it has been progressively recognised that NAFLD is a multisystem disease, where insulin resistance and related metabolic dysfunction play a pathogenic role in its development and its most relevant liver-related morbidities (cirrhosis and hepatocellular carcinoma (HCC)) (Valenzuela-Vallejo and Mantzoros, 2022). Consequently, in 2023, three large multinational liver associations proposed that metabolic dysfunction-associated steatotic liver disease (MASLD) should replace the term NAFLD (Rinella et al., 2023). The definition of MASLD is significantly different from previous diagnostic criteria for NAFLD. It requires the presence of steatotic liver disease and at least one cardiometabolic risk factor while maintaining the alcohol and concomitant liver disease exclusion criteria for NAFLD (Lee et al., 2023). This nomenclature change better reflects the pathophysiology and cardiometabolic implications of this common and burdensome liver disease. MASLD can lead to multiple clinical outcomes, including cardiovascular, metabolic, and oncologic outcomes, and may further progress to liver fibrosis, cirrhosis, and ultimately hepatocellular carcinoma (Chan et al., 2023; Ebrahimi et al., 2023). Therefore, early identification of MASLD is critical for preventing disease progression and the occurrence of complications.

Iron is essential for hemoglobin production in reticulocytes (Riley et al., 2001). Some studies have reported that iron deficiency is prevalent in patients with NAFLD, and metabolic syndrome is more common in subjects with iron deficiency (Siddique et al., 2014). Additionally, studies have suggested that metabolic disorders, such as insulin resistance, may be related to active erythropoiesis (González-Domínguez et al., 2020; Gao et al., 2022). Therefore, we reasonably inferred that reticulocytes might affect the occurrence of steatotic liver disease. However, no study has examined the potential association between reticulocytes and MASLD. In this study, we conducted cross-sectional and prospective analyses based on data from the UK Biobank to investigate the association between reticulocyte count and MASLD and identified a potential biomarker that may be suitable for predicting the development of MASLD.

## Materials and methods

### Study population and study design

This cross-sectional and prospective analysis used data from the UK Biobank. The UK Biobank is a large-scale research project that enrolled more than 500,000 participants aged 37–73 years at baseline and collected comprehensive data through the use of questionnaires, physical activity measurements, and biomarkers from blood, saliva, and urine (Rusk, 2018; Caleyachetty et al., 2021; Petermann-Rocha et al., 2022). More information on the UK Biobank protocol can be found online (http://www.ukbiobank.ac.uk). Data are available for this application. This research was conducted under the application number 92668.

### Reticulocyte counts and other covariates

Reticulocyte count was obtained from the UK Biobank Assessment Center. Reticulocyte count (10^11^ cells/L) was divided into quartiles based on the sample distribution. At the baseline assessment, age was determined using the date of birth, and self-reported data were used to determine the participants’ sex. Body mass index (BMI) was calculated from the measurements of height and weight obtained during the baseline examination. Physical exercise was classified as “yes” or “no” according to whether the metabolic equivalent task scores were more than moderate or vigorous at baseline. Participants self-reported their alcohol intake frequency at baseline, which was then classified into six categories: daily or almost daily, three to four times per week, once or twice per week, one to three times per month, special occasions only, and never. Alanine aminotransferase (ALT), aspartate aminotransferase (AST), albumin (ALB), cholesterol (CHOL), triglyceride (TG), high-density lipoprotein (HDL), low-density lipoprotein (LDL), glycated hemoglobin (HbA1c), and C-reactive protein (CRP) levels were obtained from blood samples collected at baseline.

### Definition of MASLD

We calculated the fatty liver index for each participant and defined hepatic steatosis as a fatty liver index ≥60 (Bedogni et al., 2006). MASLD was diagnosed based on hepatic steatosis and the presence of at least one of five cardiac metabolic risk factors: (1) BMI ≥25 kg/m^2^ or waist circumference >94 cm for males and >80 cm for females; (2) Fasting serum glucose ≥5.6 mmol/L or 2-h post-load glucose levels ≥7.8 mmol/L or HbA1c ≥ 5.7% or type 2 diabetes or treatment for type 2 diabetes; (3) Blood pressure ≥130/85 mmHg or specific antihypertensive drug treatment; (4) Plasma triglycerides ≥1.70 mmol/L or lipid lowering treatment; (5)Plasma HDL-cholesterol ≤1.0 mmol/L for males and ≤1.3 mmol/L for females or lipid-lowering treatment (Rinella et al., 2023).

### Statistical and subgroup analyses

Categorical variables are expressed as numbers and percentages, and continuous variables are expressed as mean ± standard deviation. Logistic regression analysis was used to calculate the odds ratios (ORs) and 95% confidence intervals (CIs) to evaluate the association between reticulocyte count and MASLD in the cross-sectional analysis. The prospective analysis used Cox proportional hazard regression models to calculate hazard ratios (HRs) and 95% CIs. Several multivariable models were constructed with confounders in both the logistic regression and Cox proportional hazard models. Model 1 was a crude model. Model 2 was adjusted for age, sex, and BMI. Model 3 was adjusted for physical exercise and alcohol intake frequency based on Model 2. Model 4 was adjusted for AST, ALT, ALB, CHOL, TG, HDL, LDL, HbA1c, and CRP levels based on Model 3. The nonlinear association between reticulocyte count and MASLD was investigated using penalized cubic splines fitted for Cox proportional hazard models. All tests were two-sided, and statistical significance was set at *p* < 0.05.

We conducted subgroup analyses based on Model 4 in both the cross-sectional and prospective analyses. Participants were clustered into groups according to sex, BMI, TG level, and fibrosis-4 index (FIB-4). The non-overweight group was defined as those with a BMI <25 kg/m^2^, and the overweight group was defined as those with a BMI ≥25 kg/m^2^. Individuals were categorized into a low TG group (TG < 2.3 mmol/L) and a high TG group (TG ≥ 2.3 mmol/L) according to TG levels (Stone et al., 2013). Liver cirrhosis was defined by the FIB-4 score and categorized as non-cirrhosis (FIB-4 <1.3) or cirrhosis (FIB-4 ≥1.3) (Sterling et al., 2006). The ORs and 95% CIs of the subgroup analyses are graphically presented using forest plots. Cumulative incidence curves were used to compare the incidence risk in different quartiles of reticulocyte count. All statistical analyses were performed using R (version 4.2.2; R Foundation for Statistical Computing, Vienna, Austria).

## Results

### Baseline characteristics

After excluding individuals with missing data, 310,091 participants were included in this cross-sectional study (Supplementary Figure S1). The baseline characteristics of the study participants were grouped based on whether they had MASLD or not, as shown in Table 1. A total of 118,042 participants were diagnosed with MASLD at baseline; among them, 79,189 [67.1%] were males, and 38,853 [32.9%] were females. The mean age of participants with MASLD was 57.08 (±7.86) years, which was significantly higher than those without MASLD (*p* < 0.001). Participants with MASLD had higher levels of BMI, ALT, AST, CHOL, TG, LDL, HbA1c, CRP, and reticulocyte count whereas the ALB and HDL levels were lower. Additionally, participants with MASLD also engaged in less physical exercise and a higher frequency of alcohol consumption.

**TABLE 1:** Characteristics of the population.

Variable	Overall (*n* = 310,091)	Non-MASLD (*n* = 192,049)	MASLD (*n* = 118,042)	*P* value
Sex, n (%)				
Female	160,151 (51.6)	121,298 (63.2)	38,853 (32.9)	<0.001
Male	149,940 (48.4)	70,751 (36.8)	79,189 (67.1)	
Age (years), mean (SD)	56.33 (8.12)	55.87 (8.24)	57.08 (7.86)	<0.001
BMI (kg/m²), mean (SD)	27.32 (4.68)	24.88 (2.80)	31.29 (4.39)	<0.001
Physical exercise, n (%)				
No	141,787 (45.7)	80,428 (41.9)	61,359 (52.0)	<0.001
Yes	168,304 (54.3)	111,621 (58.1)	56,683 (48.0)	
Alcohol intake frequency, n (%)				
Never	66,021 (21.3)	40,349 (21.0)	25,672 (21.7)	<0.001
Special occasions only	74,357 (24.0)	47,426 (24.7)	26,931 (22.8)	
1-3 times a month	80,092 (25.8)	50,186 (26.1)	29,906 (25.3)	
Once or twice a week	33,884 (10.9)	20,913 (10.9)	12,971 (11.0)	
3-4 times a week	32,928 (10.6)	19,617 (10.2)	13,311 (11.3)	
Daily or almost daily	22,809 (7.4)	3,558 (7.1)	9,251 (7.8)	
ALT (U/L), mean (SD)	23.59 (14.02)	19.45 (9.44)	30.33 (17.26)	<0.001
AST (U/L), mean (SD)	26.20 (9.97)	24.57 (7.92)	28.86 (12.17)	<0.001
ALB (g/L), mean (SD)	45.26 (2.61)	45.30 (2.59)	45.19 (2.64)	<0.001
CHOL (mmol/L), mean (SD)	5.69 (1.13)	5.67 (1.08)	5.72 (1.21)	<0.001
TG (mmol/L), mean (SD)	1.74 (1.01)	1.34 (0.62)	2.40 (1.18)	<0.001
HDL (mmol/L), mean (SD)	1.45 (0.38)	1.57 (0.37)	1.25 (0.29)	<0.001
LDL (mmol/L), mean (SD)	3.56 (0.86)	3.51 (0.83)	3.64 (0.91)	<0.001
HbA1c (%), mean (SD)	5.44 (0.60)	5.34 (0.44)	5.61 (0.75)	<0.001
CRP (mg/L), mean (SD)	2.51 (4.20)	1.90 (3.72)	3.49 (4.72)	<0.001
Reticulocyte counts (×10^11^ cells/L), mean (SD)				
quartile 1	80,955 (26.1)	69,417 (36.1)	11,538 ( 9.8)	<0.001
quartile 2	79,469 (25.6)	54,978 (28.6)	24,491 (20.7)	
quartile 3	75,497 (24.3)	40,808 (21.2)	34,689 (29.4)	
quartile 4	74,170 (23.9)	26,846 (14.0)	47,324 (40.1)	

MASLD: metabolic dysfunction-associated steatotic liver disease; SD: standard deviation; TG: triglyceride; BMI: body mass index; ALT: alanine aminotransferase; AST: aspartate aminotransferase; ALB: albumin; CHOL: cholesterol; TG: triglyceride; HDL: high-density lipoprotein; LDL: low-density lipoprotein; HbA1c: glycated hemoglobin; CRP: C-reactive protein.

### Cross-sectional analysis

**FIGURE 1:**
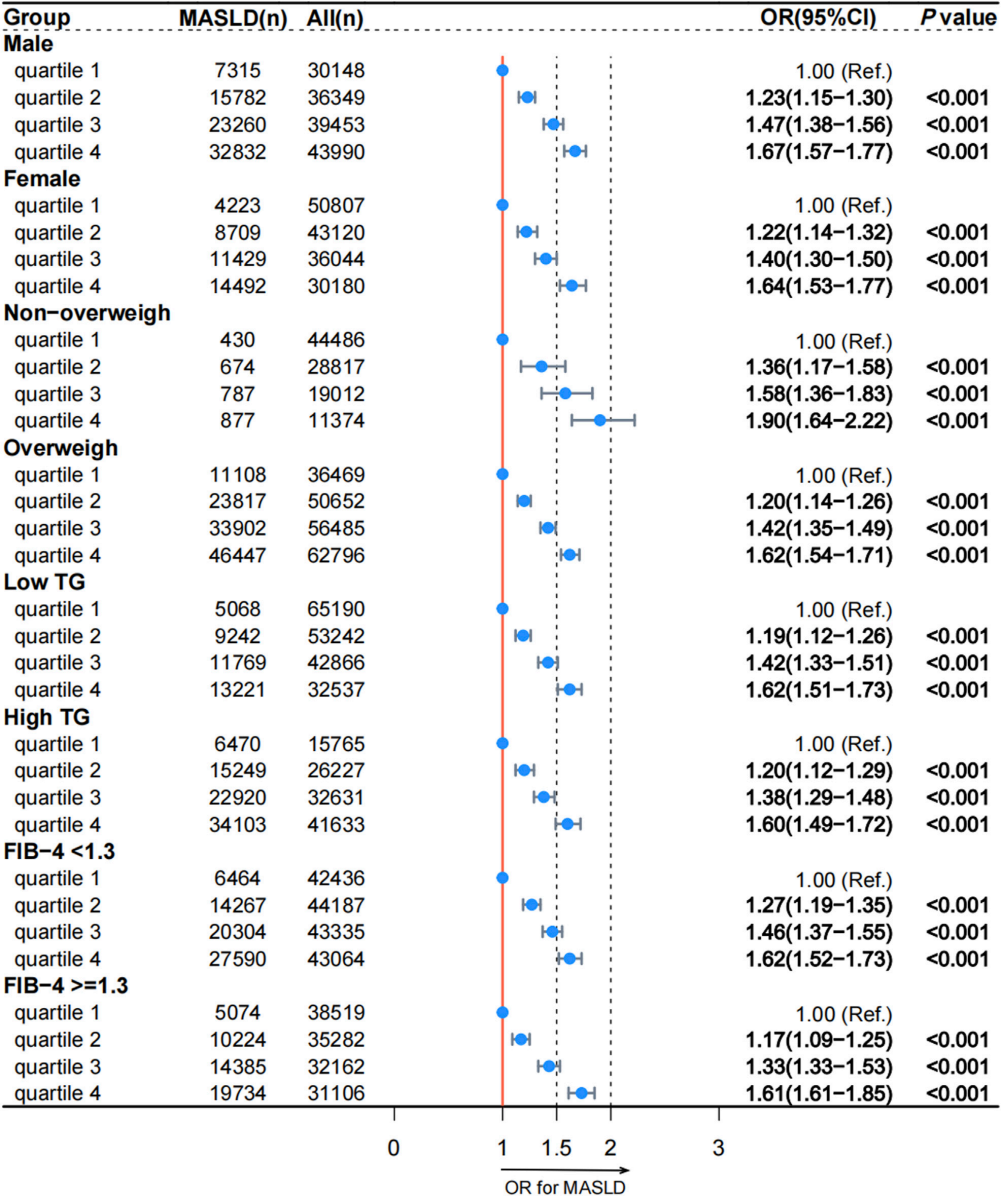
Association between reticulocyte count quartiles and MASLD in subgroups according to the cross-sectional analysis. MASLD: metabolic dysfunction-associated steatotic liver disease; TG: triglyceride; FIB-4: fibrosis-4; OR: odds ratio; CI: confidence interval.

The results of the logistic regression analysis of the association between reticulocyte count and MASLD across the various models are shown in Table 2. A significant positive association was observed between increasing reticulocyte count quartiles and the presence of MASLD in all models. In Model 4, the adjusted ORs for quartiles 2, 3, and 4 of reticulocyte count with MASLD were 1.22 (95% CI, 1.17–1.28; *p* < 0.001), 1.44 (95% CI, 1.38–1.51; *p* < 0.001) and 1.66 (95% CI, 1.59–1.74; *p* < 0.001), respectively. Subgroup analyses based on sex, BMI, TG levels, and the FIB-4 index were conducted to verify the sensitivity of reticulocyte counts, and the results were shown in Figure 1. After adjusting for the covariates, the association between reticulocyte count quartiles and MASLD in all subgroups remained significant. Supplementary Figure S2 illustrates the nonlinear relationship between reticulocyte counts and MASLD. Across all subgroups and after adjusting for covariates, higher reticulocyte counts levels were significantly associated with an increased risk of MASLD (*p* < 0.001).

**TABLE 2:** ORs (95% CIs) of MASLD across quartiles of reticulocyte count.

	Reticulocyte count	Model 1			Model 2			Model 3			Model 4		
		OR	95% CI	*P* value	OR	95% CI	*P* value	OR	95% CI	*P* value	OR	95% CI	*P* value
MASLD	Quartile 1	—	—		—	—		—	—		—	—	
	Quartile 2	2.68	2.61–2.75	<0.001	1.71	1.65, 1.77	<0.001	1.69	1.63, 1.75	<0.001	1.22	1.17–1.28	<0.001
	Quartile 3	5.11	4.99–5.24	<0.001	2.52	2.43, 2.61	<0.001	2.47	2.39, 2.56	<0.001	1.44	1.38–1.51	<0.001
	Quartile 4	10.6	10.3–10.9	<0.001	3.96	3.82, 4.11	<0.001	3.87	3.73, 4.02	<0.001	1.66	1.59–1.74	<0.001

OR: odds ratio; CI: confidence interval; MASLD: metabolic dysfunctional associated fatty liver disease.

### Prospective analysis

**FIGURE 2:**
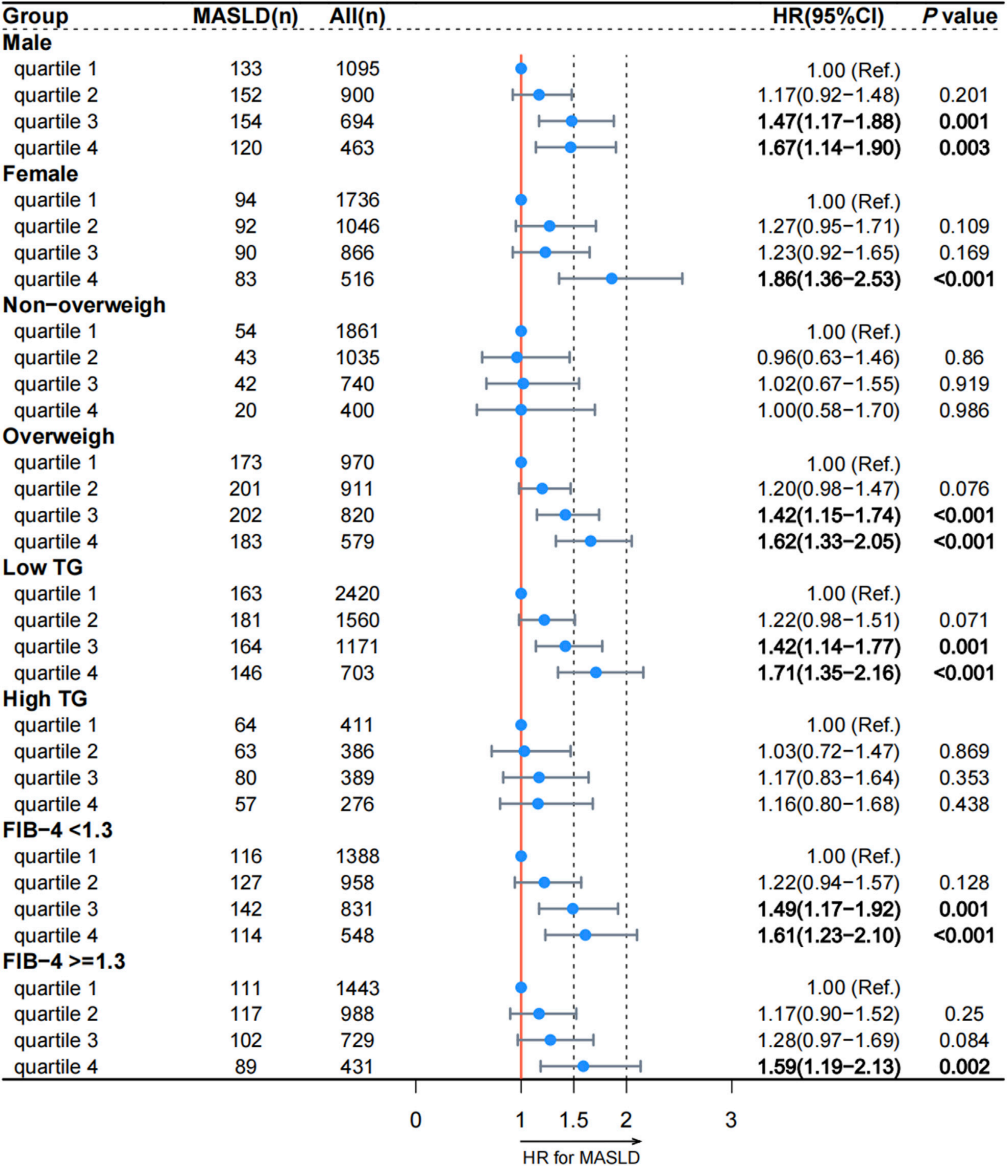
Association between reticulocyte count quartiles and MASLD in subgroups according to the prospective analysis. MASLD: metabolic dysfunction-associated steatotic liver disease; TG: triglyceride; FIB-4: fibrosis-4. HR: hazard ratio; CI: confidence interval.

**FIGURE 3:**
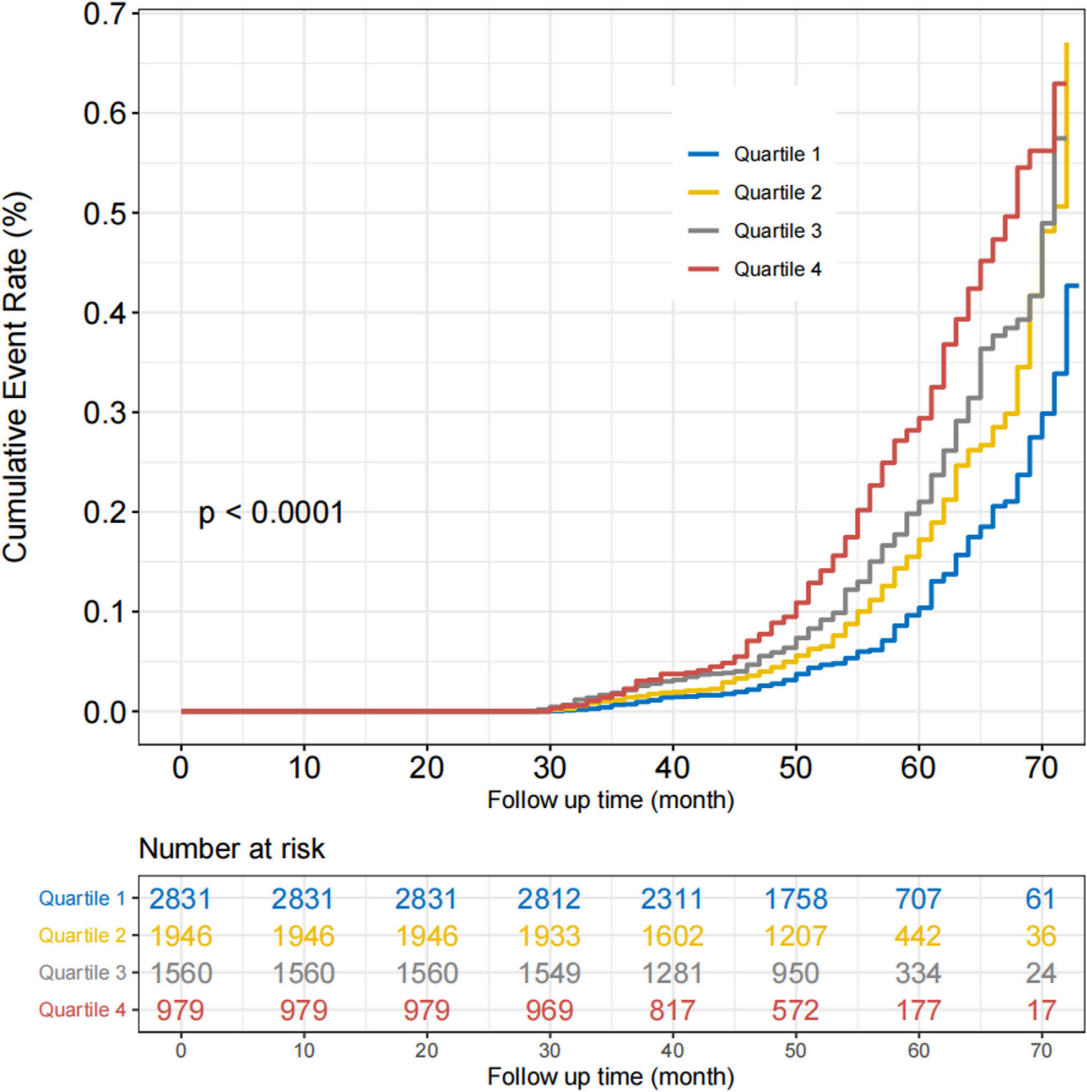
Cumulative incidence rate curve of MASLD with different reticulocyte count quartiles.

We conducted a prospective analysis to further explore the relationship between reticulocyte count and MASLD. After excluding those without follow-up data, 7,316 participants without MASLD were included in this prospective study (Supplementary Figure S1). The Cox regression analysis was used to examine the association between reticulocyte count and MASLD, and the results are presented in Table 3. In Model 4, the adjusted HRs for quartiles 2, 3, and 4 of MASLD were 1.19 (95% CI, 0.99–1.43; *p* = 0.063), 1.37 (95% CI, 1.14–1.65; *p* < 0.001), and 1.55 (95% CI, 1.28–1.89; *p* < 0.001), respectively. After adjusting for covariates, most subgroups had the similar results, as shown in Figure 2. In all subgroups except for high TG and non-overweight, quartiles 4 of reticulocyte count was associated with a higher risk of MASLD. The curve of the incidence rate according to the different quartiles of reticulocyte count is shown in Figure 3. The results demonstrated a significant trend, where higher reticulocyte count quartiles were associated with an increased incidence of MASLD (*p* < 0.001).

**Table 3:** HRs (95% CIs) of MASLD across quartiles of reticulocyte count in prospective cohort.

	Reticulocyte counts	Model 1			Model 2			Model 3			Model 4		
		HR	95% CI	*P* value	HR	95% CI	*P* value	HR	95% CI	*P* value	HR	95% CI	*P* value
MASLD	Quartile 1	—	—		—	—		—	—		—	—	
	Quartile 2	1.64	1.37–1.96	<0.001	1.26	1.05–1.51	<0.001	1.26	1.05–1.51	0.012	1.19	0.99–1.43	0.063
	Quartile 3	2.10	1.75–2.51	<0.001	1.56	1.30–1.87	<0.001	1.56	1.30–1.86	<0.001	1.37	1.14–1.65	<0.001
	Quartile 4	3.03	2.50–3.66	<0.001	1.84	1.52–2.22	<0.001	1.83	1.51–2.22	<0.001	1.55	1.28–1.89	<0.001

HR: hazard ratio; CI: confidence interval; MASLD: metabolic dysfunctional associated fatty liver disease.

## Discussion

This study, based on data from a substantial cohort of 310,091 participants from the UK Biobank, represents the first exploration of the association between reticulocyte counts and MASLD. The study followed a two-phase approach. Initially, a retrospective analysis revealed a positive association between reticulocyte count and MASLD. Subsequently, a prospective study involving patients without MASLD indicated that higher baseline reticulocyte counts were positively associated with the development of MASLD. These findings collectively suggest that elevated reticulocyte levels constitute an independent risk factor for MASLD.

Previous studies have identified potential hematological markers that can predict fatty liver diseases. Michalak et al. performed a retrospective study and found that red blood cell distribution width, platelet ratio, and lymphocyte ratio were significantly higher in the metabolic-associated fatty liver disease group (Michalak et al., 2022). Another study showed that an increased red blood cell count is linked to a greater likelihood of metabolic-associated fatty liver disease in males (Dai et al., 2022). One study revealed an independent association between high red blood cell count and an increased risk of both the incidence and progression of NAFLD (Zhong et al., 2021). Our study benefited from a substantially larger sample size compared to prior research, enhancing the precision and reliability of our findings. To the best of our knowledge, this is the first study to explore the relationship between reticulocyte count and MASLD.

While the exact mechanism underlying the connection between reticulocytes and MASLD remains largely uncharted, prior research provides some insights. Iron is an essential element in all living organisms and is required as a cofactor for oxygen-binding proteins. Iron metabolism, oxygen homeostasis and erythropoiesis are consequently strongly interconnected (Peyssonnaux et al., 2008). Iron plays a crucial role in hemoglobin production, and sufficient iron levels can increase reticulocyte count (Newhall et al., 2020). Previous studies have shown that iron status indicators were significantly positively associated with fatty liver disease (Tan et al., 2023). A cross-sectional study, involving 5445 Chinese individuals, showed that there was a dose-response relationship between dietary iron intake and the prevalence of MAFLD (Yang et al., 2019). In a case-control study in Southeast China, Pan et al. also found that elevated serum ferritin levels were associated with a higher risk of MAFLD (adjusted-odds ratio 1.62, 95% CI 1.16–2.27) (Pan et al., 2019). Therefore, we believe that the results of this study can be attributed to iron metabolism. The results of this study can also be explained by insulin resistance. Insulin resistance is defined clinically as the inability of a known quantity of exogenous or endogenous insulin to increase glucose uptake and utilization in an individual as much as it does in a normal population (Lebovitz, 2001). Recently, researchers have found that insulin resistance plays pivotal role in liver steatosis and even more so in steatohepatitis (Marušić et al., 2021). Insulin resistance enhances the expression and activity of hormone-sensitive lipase (HSL) in adipose tissue, and HSL can catalyze the hydrolysis of TG to free fatty acid (FFA) (Yanai et al., 2023; Fisher, 2012). Insulin resistance reduces fatty acid (FA) oxidation, leading to diminished use of FAs and storage of TG within the skeletal muscle (Yanai et al., 2023). Serum FFAs increase due to increased release from the adipose tissue and decreased FA use in the skeletal muscle (Yanai et al., 2023). Excessive FFA is essential to start the vicious self-perpetuating circle of NAFLD (Marušić et al., 2021). Additionally, insulin resistance is also related to reticulocytes. As supported by both cross-sectional and longitudinal analyses, young adults with insulin resistance are more likely to have increased red blood cell counts and haematocrit levels, even in the presence of normal glucose levels (Ferreira et al., 2019). *In vitro* evidence suggests that hyperinsulinemia may involve multiple pathways that affect erythropoiesis. For instance, insulin and its analogs, such as insulin-like growth factor-1, can work in conjunction with erythropoietin to promote erythroid colony proliferation (Aoki et al., 1994; Miyagawa et al., 2000). These mechanisms may explain the relationship between reticulocytes and MASLD.

Despite the significant findings uncovered, this study also has some limitations. First, owing to the unavailability of hepatic imaging or biopsy data, we used the fatty liver index to diagnose MASLD. Although fatty liver index is a simple and commonly accepted indicator of human fatty liver disease in the absence of imaging and histological data (Liu et al., 2022; Wang et al., 2024), liver biopsy is the gold standard for diagnosing liver steatosis. Second, despite accounting for an extensive array of confounding factors in our analysis, the possibility of unmeasured or residual confounding factors persisted. Finally, this study lacked data from the Asian population. Further prospective studies are required to better understand the precise relationship between reticulocytes and MASLD.

In conclusion, we found evidence that increased reticulocyte count is independently associated with a higher risk of MASLD. Our findings suggest that reticulocyte count can potentially be used as a large-scale noninvasive tool to monitor the occurrence and development of MASLD dynamically.

## Data Availability

Publicly available datasets were analyzed in this study. This data can be found here: http://www.ukbiobank.ac.uk/.

## Supplementary material

SUPPLEMENTARY FIGURE S1 Flowchart of the patients included in the study. MASLD: metabolic dysfunction-associated steatotic liver disease.

SUPPLEMENTARY FIGURE S2 Nonlinear association between reticulocyte count and MASLD by subgroup according to the cross-sectional analysis using penalized cubic splines. MASLD: metabolic dysfunction-associated steatotic liver disease. **(A)** male group; **(B)** female group; **(C)** non−overweigh group; **(D)** overweigh group; **(E)** low TG group; **(F)** high TG group; **(G)** FIB-4 <1.3 group; (H) FIB-4 ≥1.3 group.

## Abbreviations

ALB, albumin; ALT, alanine aminotransferase; AST, aspartate aminotransferase; BMI, body mass index; CHOL, cholesterol; CI, confidence interval; CRP, C-reactive protein; FIB-4, fibrosis-4; HbA1c, glycated hemoglobin; HDL, high-density lipoprotein; HR, hazard ratio; LDL, low-density lipoprotein; MASLD: metabolic dysfunction-associated steatotic liver disease; NAFLD, nonalcoholic fatty liver disease; OR, odds ratio; TG, triglyceride.
