# Supplementary figures and images for: A high reticulocyte count is a risk factor for the onset of metabolic dysfunction-associated steatotic liver disease: Cross-sectional and prospective studies of data of 310,091 individuals from the UK Biobank
Source: Front Pharmacol. 2024 Jul 1;15:1281095. doi: 10.3389/fphar.2024.1281095 (PMC11247344; doi:10.3389/fphar.2024.1281095)

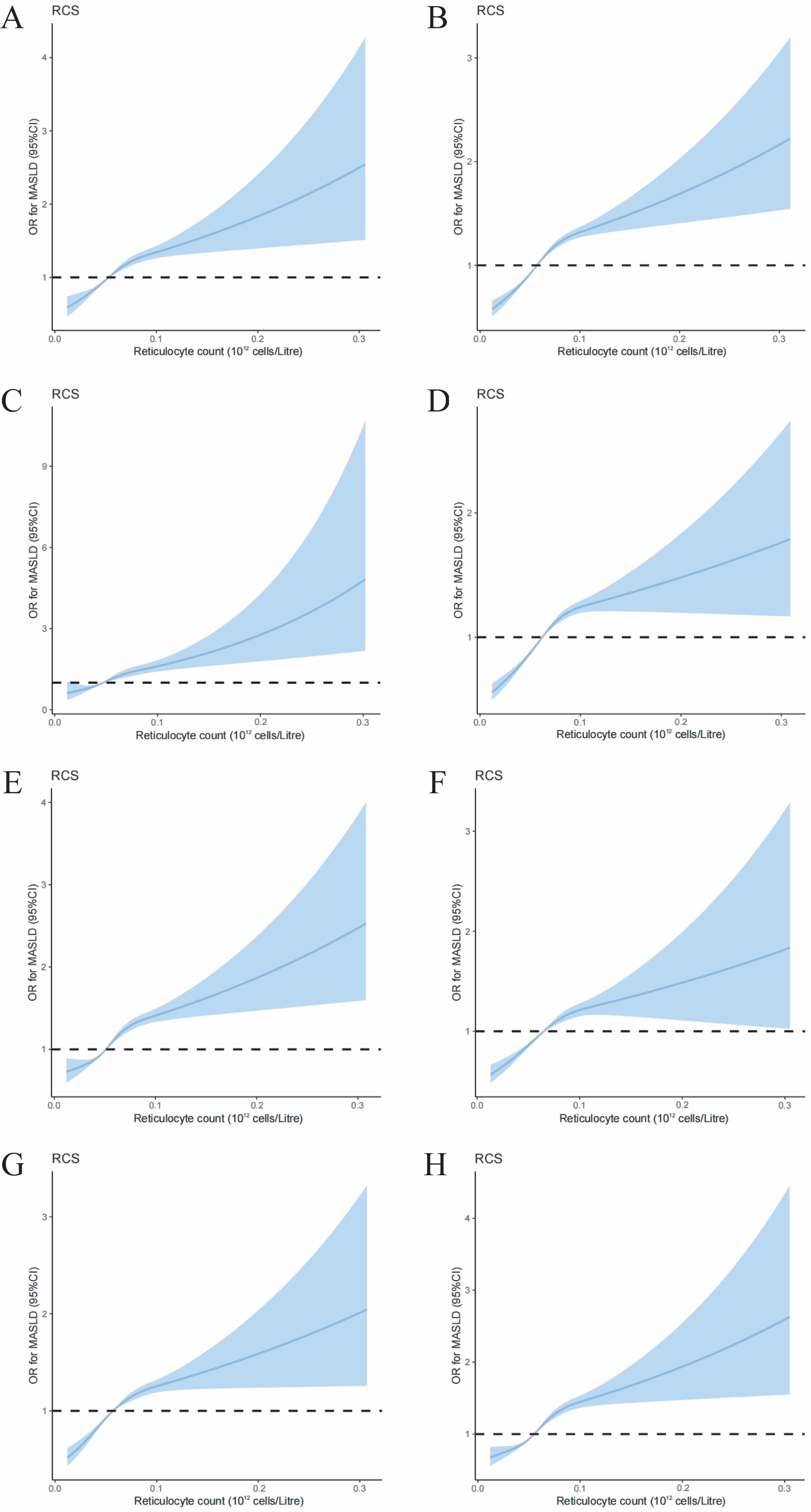

Supplement: Supplementary file 1 [file Image2.jpg]

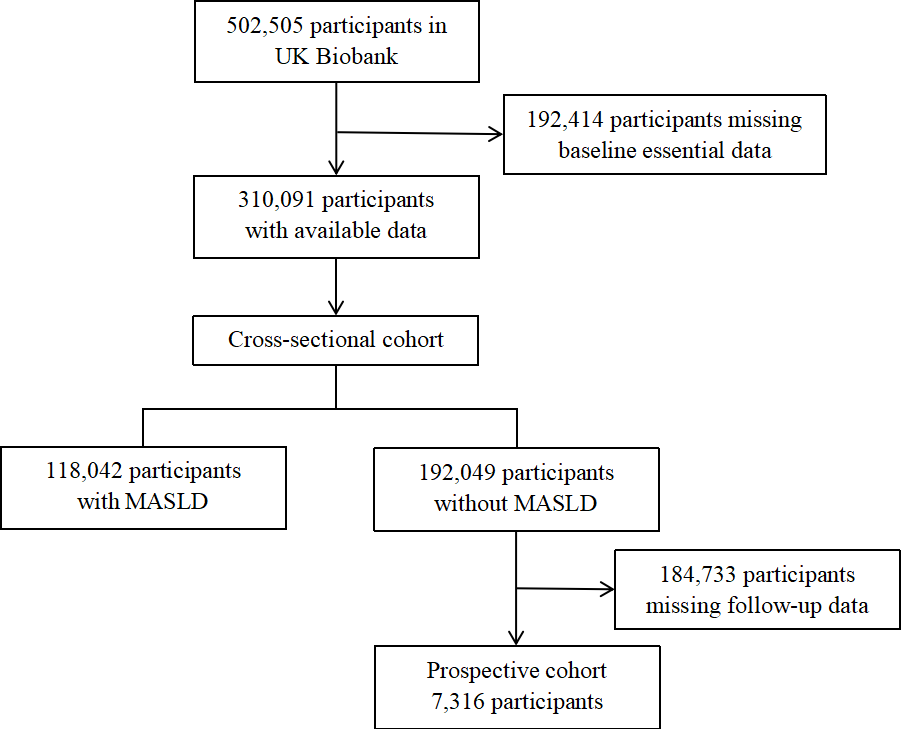

Supplement: Supplementary file 2 [file Image1.TIF]
